# Antiviral therapy for chronic hepatitis B in China

**DOI:** 10.1007/s00430-014-0380-z

**Published:** 2014-12-25

**Authors:** Xin Zheng, Junzhong Wang, Dongliang Yang

**Affiliations:** Department of Infectious Disease, Union Hospital, Tongji Medical College, Huazhong University of Science and Technology, Jiefang Avenue 1277, Wuhan, China

**Keywords:** Chronic hepatitis B, Antiviral therapy, Drug resistance, China

## Abstract

The vaccination program against hepatitis B virus (HBV) has greatly reduced the incidence of HBV infection. However, almost one-fourth of the HBV infected patients worldwide are still located in China. The healthcare burden from chronic HBV infection is a big challenge for the Chinese government and clinicians. Antiviral therapy plays a central role in controlling chronic HBV infection and preventing the disease progression. However, due to the specific economic and medical system issues, the first-line antiviral agents recommended by the AASLD and EASL have not been widely used for Chinese patients. In this review, we will discuss some key issues in the area of antiviral treatment for chronic hepatitis B in China.

## Introduction

There are about 350 million people worldwide suffering from chronic hepatitis B virus (HBV) infection. China is one of the countries with a high prevalence of HBV infection [1]. The HBV vaccination program for all newborns was implemented by the Chinese government in 1992, which increased the yearly national HBV vaccine inoculation rate. Based on this achievement, the prevalence of HBV surface antigen (HBsAg) carrier in the general population (1–59 years old) of China was decreased from 9.75 % in 1992 to 7.18 % in 2006 [1]. Despite the reduction in the incidence of HBV infection, there are still about 93 million people chronically infected with HBV and an estimated 20 million cases of symptomatic chronic hepatitis B in China now. Therefore, the healthcare burden from chronic HBV infection is still a big challenge for China.

Patients with chronic HBV infection may develop liver fibrosis, cirrhosis, hepatic decompensation, and hepatocellular carcinoma (HCC) and eventually die from liver failure and other complications [2]. Numerous studies have shown that active HBV replication leads to liver injury and disease progression [3]. As pointed out by the “Guidelines for prevention and treatment of chronic hepatitis B” from the American Association for the Study of liver disease (AASLD), European Association for the Study of the Liver (EASL), Asia Pacific Association for the Study of the Liver (APASL), and Chinese Association for the Study of liver disease (CASLD), the ultimate long-term goal of treatment is to prevent hepatic decompensation, reduce progression to cirrhosis and/or HCC, and prolong survival [4]. The short-term goal is to permanently suppress HBV replication. Therefore, antiviral therapy plays a central role in controlling chronic HBV infection and preventing the disease progression. Due to economic and medical system issues, the antiviral treatment in China has its own unique characteristics [5]. In this review, we will discuss some key issues in the area of antiviral treatment for chronic hepatitis B in China.

## Patients who need antiviral therapy

Patients with positive serum HBsAg for more than 6 months or patients with positive HBsAg and chronic liver disease proven by biopsy can be diagnosed with chronic HBV infection. Based on the information from the guidelines for management of chronic hepatitis B virus infection published by CASLD in 2010, the indications for antiviral therapy are as follows:

General indications: (1) For HBeAg positive patients with serum HBV DNA ≥ 10^5^ copies/mL (equivalent to 20,000 IU/mL) and for HBeAg negative patients with HBV DNA ≥ 10^4^ copies/mL (equivalent to 2,000 IU/mL); (2) alanine transaminase (ALT) ≥ 2 upper limit of normal (ULN), but for patients treated with interferon-α (IFN-α), ALT should be ≤10 ULN and the total bilirubin should be <2 ULN;and (3) ALT < 2 ULN, but the liver biopsy shows a histological activity index (Knodell HAI) ≥4, or degree of necroinflammation ≥G2, or degree of fibrosis ≥S2.

For patients with persistently positive HBV DNA who do not show the criteria mentioned above, the antiviral therapy should be applied if they are in the following conditions:

1. Patients older than 40 years and ALT > ULN.
2. For patients older than 40 years and ALT is sustained normal, a liver biopsy is recommended. If the Knodell HAI is ≥4, or the degree of necroinflammation ≥G2, or the degree of fibrosis ≥S2, then antiviral therapy should be applied.
3. If disease progression is evident, for example, spleen enlargement, liver biopsy is recommended, and antiviral therapy should be applied if necessary [1].

As to the standards for determining the patients who need antiviral therapy, CASLD has very similar rules as AASLD, EASL, or APASL. All these associations recommend that the patients need to be evaluated based on the state of persistent HBsAg, HBeAg, the level of HBV DNA, and the degree of liver inflammation and fibrosis, and clinicians may decide whether the patients are appropriate for antiviral therapy. Histology of the liver biopsy provides direct evidence for grading the inflammation and staging the fibrosis in the liver; however, liver biopsy is hard to perform in most hospitals because of the patients’ refraction.

ALT is routinely tested as a biochemical marker to reflect the degree of the liver inflammation, it is one of the important markers for identifying whether the patient in an immune activation stage or not. The ULN for ALT is defined as 40U/L in China based on the results of a national wide survey in the 1950s [6, 7]. However, increasing evidence from China shows that this reference value cannot dependably reflect the degree of the liver inflammation. For chronic hepatitis B patients with persistently normal ALT, the liver biopsy results showed that 34.4 % of them had fibrosis with degree ≥S2 or fibrosis degree ≥S1 and inflammation degree ≥G2 [8]. Another study reported that in patients with ALT < 0.5 ULN, the proportion with significant liver inflammation and fibrosis was 16.6 %, whereas in patients with ALT between 0.5 and 1 ULN, the proportion increased to 40 % [9]. For patients with ALT between 1 ULN and 2 ULN, the proportion of fibrosis could be up to 61.8 % [8]. These data indicate that the ULN of ALT may need to be revised for China.

A large sample survey in Italy showed the ULN of ALT should be 30 U/L for healthy men and 19 U/L for healthy women [10]. The guidelines of AASLD in 2009 recommended to use these values to evaluate the liver inflammation status of the patients [11]. A study in Korea showed that the ULN of ALT for Koreans was 33 U/L for the healthy men and 25 U/L for healthy women [12], and these values had been applied in the guidelines for Korea in 2012 [13]. A large sample survey for ULN of ALT, including 28,642 healthy men and 24,413 healthy women was performed in China in 2011 and showed the ULN of ALT was 35 U/for men and 23 U/L for women in Chinese Han population [14]. Based on these new findings, CASLD should adjust the ULN of ALT reference value in the next version of the guidelines. Otherwise, the patients with mildly elevated ALT (ALT between 1 ULN and 2 ULN) or persistently normal ALT may not receive the opportunity of antiviral therapy.

## Drugs available for antiviral therapy in China

Two kinds of antiviral agents have been approved for the therapy of chronic hepatitis B: IFN-α and nucleot(s)ide analogs. The first includes conventional IFN-α and Peg-IFN-α, and the second includes lamivudine (LAM), adefovir dipivoxil (ADV), telbivudine (LdT), entecavir (ETV), and tenofovir (TFV). All these medicines are now available in China. The randomized clinical trials confirmed that Peg-IFN-α treatment could obtain a higher HBeAg seroconversion rate in a limited period of treatment; ETV and TFV have a potent antiviral effect, low incidence of drug resistance, and good safety [11, 15–19]. These drugs are listed as the first-line antiviral drugs for chronic hepatitis B treatment by AASLD and EASL. The conventional IFN-α, LdT, ADV, and LAM are the second-line antiviral drugs [11, 20]. In China, compared with the average yearly incomes of the general population, the Peg-IFN-α and ETV produced by foreign or Sino-foreign joint venture pharmaceutical companies are very expensive and are only covered by a small proportion of medicare payments, or not covered at all. Most of Chinese patients with chronic hepatitis B cannot afford Peg-IFN-α or ETV, and consequently these drugs are rarely used for the initial antiviral treatment. The clinicians have to choose from the second-line drugs with affordable prices for the patients, especially in rural areas. By using the second-line drugs, the health system reduces the cost of treatment in a short term, but the medical expense will increase in the long term if the patients develop drug resistance or suboptimal response.

With the development of local pharmaceutical companies, the generic drugs of conventional IFN-α, LAM, ADV, and ETV have been successfully produced and approved for the treatment of chronic hepatitis B in China. The application of domestic produced Peg-IFN-α in chronic hepatitis B is still in clinical trial. The multicenter, large sample clinical studies show that there are no significant differences in antiviral efficacy, bioavailability, and safety between the domestic IFN-α/nucleot(s)ide analogs and the original drugs [21–23]. The price of domestic generic drugs is only 50–70 % of the original drugs. This provides more choices for the clinician in addition to reducing the economic pressure on the patients.

Traditional Chinese medicines (TCM) have been used for treating chronic hepatitis B for a long time. Over 90 % of chronic hepatitis B patients received the TCM therapy [24]. Preclinical and clinical trials, which were performed in China, indicated that some traditional Chinese medicine preparations, like phyllanthus urinaria, decoction of small bupleurum (a medicinal root found natively in East Asia), matrine (an alkaloid found in plants from the Sophora genus with anticancer activity), or rhubarb, had a potential effect on inhibiting HBV replication, regulating the hosts’ immune function, and improving liver function [25, 26]. A short-term clinical follow-up study found that matrine treatment could result in a similar curative effect as interferon in HBeAg seroconversion and HBV DNA reduction [27]. The small bupleurum decoction and phyllanthus urinaria preparations are reported to have a similar therapeutic effect as conventional IFN [26, 28]. Although it seems that TCM can be used as alternative medicine for antiviral treatment, the antiviral effect needs to be evaluated in well-designed and randomized clinical trials. In addition, the composition of TCM is very complex, and it is difficult to explain the mechanism from the standpoint of modern medicine. This limits the application of TCM in the treatment of chronic hepatitis B [25, 29].

## The drug resistance and rescue strategies

Due to the low cost, the second-line nucleot(s)ide analogs (LAM, LdT, and ADV) are widely used in China. When the second-line NAs are chosen for long-term therapy regimens, drug resistance may occur and cause virologic breakthrough, hepatitis flare, and even death. Therefore, rescue strategies need to be taken once the resistant strains of HBV are detected. For the LAM-resistant strain, the therapy can switch to monotherapy with either Adefovir or Entecavir, add-on ADV to LAM or use a combination treatment of ADV and ETV. The experience from China is that ETV + ADV is superior to LAM + ADV, and LAM + ADV is better than monotherapy [30, 31]. However, due to the risk of frequent resistance development against ETV with LAM-resistant strains, further research is needed to assess long-term cost-effectiveness of ETV combination treatment to LAM-resistant disease. For the ADV-resistant strains, adding LAM, or LdT, or ETV to ADV is recommended [1]. Current research shows that both LdT + ADV combination therapy and ETV monotherapy leads to significant decreases in serum HBV DNA in the patients with resistance to ADV, and LdT + ADV combination therapy exhibits a significantly higher rate of HBeAg seroconversion than ETV monotherapy [32]. For the patients with resistance to LdT or ETV, adding ADV is recommended, but its antiviral efficacy and safety are not established [1]. A small sample survey showed that LdT + ADV combination therapy led to significant decreases in serum HBV DNA levels, normalization of ALT, and increased the rate of HBeAg seroconversion in patients with resistance to LdT [33].

## Response guided therapy

The serum HBV DNA levels, serum ALT levels, HBV genotype, HBeAg status, and other factors in patients with initial antiviral treatment are important factors for predicting the response to antiviral therapy which has been called baseline guided therapy (BGT). In recent years, the response of patients to treatment during antiviral treatment as the guidance (response guided therapy, RGT) has become the focus of attention. A multicenter study on optimization of telbivudine (LdT) treatment led by Hou in China is based on the idea of RGT. Chronic hepatitis B patients were evaluated after 24 weeks of LdT monotherapy. If the serum level of HBV DNA was ≥300 copies/mL, patients were switched to LdT plus ADV combination therapy. Otherwise, patients would maintain LdT monotherapy. After 105 weeks of treatment, the rates of HBV DNA < 300 copies/mL in the LdT plus ADV group and the LdT group were not significantly different (76.7 vs. 61.2 %). However, the rates of viral mutations and drug resistance were significantly different between these two groups (6.0 vs. 30.4 %, 2.7 vs. 25.8 %) [34].

## Combination therapy

Combination antiviral therapy strategy has been used for rescue therapy and RGT. It is uncertain whether it should be used for initial treatment. Some studies indicated that initial combination antiviral treatment can improve efficacy and reduce the rates of viral mutations in HBeAg positive CHB patients with high viral load (≥10^8^ IU/mL) and in decompensated cirrhosis patients who need long-term NAs antiviral therapy [35, 36]. The combination of IFN-α and NA or two NAs are the mainstream strategy for combination treatment. Although there are some reports from China showed that combination treatment was significantly superior to monotherapy (see Table 1) [37]. However, the CASLD does not recommend the initial LAM-ADV combination therapy for CHB patients.

**Table 1:** Results of initial combination antiviral therapy for chronic hepatitis B

Groups	Virologic response %	HBeAg clearance %	HBeAg seroconversion %	HBsAg clearance %	ALT normalization %	Drug resistance %	Time (weeks)	Number of patients	References
LAM + ADV	100	–	51	–	100	0	96	50	He et al. [38]
LAM	66	–	21	–	77	34	96	50	
ADV	49	–	33	–	64	4	96	50	
Ldt + ADV	90.6	46.9	37.5	–	93.8	0	96	32	Li et al. [39]
LAM + ADV	68.8	21.9	15.6	–	87.5	0	96	32	
Peg-IFN-α-2a + ETV	100	30	10	–	90	–	24	20	Zeng et al. [40]
Peg-IFN-α-2a	30	15	0	–	50	–	24	20	
ETV	90	20	5	–	85	–	24	20	
Peg-IFN-α-2a + ADV	48.6	–	45.7	–	60	–	96	35	Ma et al. [41]
Peg-IFN-α-2a	34.2	–	42.1	–	60.5	–	96	38	
ADV	12.5	–	15	–	37.5	–	96	40	
Peg-IFN-α-2a + LAM	100	80	75	35	–	–	96	20	Cao et al. [37]
Peg-IFN-α-2a + ADV	100	76	71	24	–	–	96	21	

– means that no data were reported

TCM are also candidates for combination therapy. The combination of matrine and nucleot(s)ide analogs, such as LAM, has synergistic effect and can achieve even better therapeutic effects for inhibiting HBV replication. They can restore liver function quickly and reduce the incidence of virus bounce after withdrawal of the nucleot(s)ide analogs [25, 27]. But it need further study to confirm.

## Prospective

It is clear that many chronic HBV patients have benefited from antiviral treatment. The incidence of liver fibrosis, cirrhosis, hepatic decompensation, and HCC has been reduced. However, the clinicians have new challenges to face. In the US and European countries, primary non-response and suboptimal response is rare in patients who received TDF and ETV treatment, but may occur due to pharmaceutical response of the host to the drugs. In China, poor adherence of patients to treatment, relative high costs of the first-line antiviral drugs and resistance development may be the key factors that cause the non-response, suboptimal response, and even treatment failure. Since 2008, several large cohort studies supported by the government have been carried out on the optimizing strategy for the treatment of chronic hepatitis B in China. This may serve as one of the important ways to solve this problem and to benefit most of the patients. In addition, recent studies show that the agonist of Toll-like receptor 7 and HBsAg/HBsAb immune complex can induce prolonged suppression of hepatitis B virus in chronically infected individules by stimulating the innate and acquired immune response. Therefore, the immune modulation therapy may be a new strategy for antiviral treatment on chronic hepatitis B.
